# Oxidative stress and antioxidant defense responses in *Acartia* copepods in relation to environmental factors

**DOI:** 10.1371/journal.pone.0195981

**Published:** 2018-04-13

**Authors:** Olivier Glippa, Jonna Engström-Öst, Mirella Kanerva, Anni Rein, Kristiina Vuori

**Affiliations:** 1 Novia University of Applied Sciences, Ekenäs, Finland; 2 Laboratory of Animal Physiology, Department of Biology, University of Turku, Turku, Finland; Universidade de Brasilia, BRAZIL

## Abstract

On a daily basis, planktonic organisms migrate vertically and thus experience widely varying conditions in their physico-chemical environment. In the Gulf of Finland, these changes are larger than values predicted by climate change scenarios predicted for the next century (up to 0.5 units in pH and 5°C in temperature). In this work, we are interested in how temporal variations in physico-chemical characteristics of the water column on a daily and weekly scale influence oxidative stress level and antioxidant responses in the planktonic copepod of the genus *Acartia*. Responses were determined from samples collected during a two-week field survey in the western Gulf of Finland, Baltic Sea. Our results showed that GST (Glutathione-S-transferase) enzyme activity increased in the surface waters between Weeks I and II, indicating antioxidant defense mechanism activation. This is most likely due to elevating temperature, pH, and dissolved oxygen observed between these two weeks. During Week II also GSSG (oxidized glutathione) was detected, indicating that copepods responded to stressor(s) in the environment. Our results suggest that *Acartia* copepods seem fairly tolerant to weekly fluctuations in environmental conditions in coastal and estuarine areas, in terms of antioxidant defense and oxidative stress. This could be directly connected to a very efficient glutathione cycling system acting as antioxidant defense system for neutralizing ROS and avoiding elevated levels of LPX.

## Introduction

Carbon dioxide, Ultraviolet radiation (UV-B), eutrophication, hypoxia, and salinity changes are strongly modifying the pelagic and coastal marine ecosystems globally [1, 2, 3, 4]. In addition, the coastal environment can be affected by upwelling of deep-water as well as by freshwater riverine input [5]. Zooplankton, living in these habitats, are a central part of the marine food-web, between microzooplankton, primary producers and higher trophic levels, such as fish larvae, mysid shrimps and jellyfish [6]. Copepods are excellent model organisms to study as they experience large variability in their physico-chemical environment (i.e., light, temperature, salinity, pH, oxygen and chlorophyll *a* conditions) by performing diel vertical migration. Thus, Almén et al. [7] reported that copepods from a coastal area in the south-western Finland can experience a change in pH up to >0.5 units, and a change in temperature of 5°C on a daily basis, which means larger fluctuations than climate change scenarios propose for the coming century [7,8]. Moreover, the same species was found to experience a change in salinity of 0.24 [7]. The migration behavior is likely to affect the physiological plasticity in copepods and can have positive effects on their ability to cope with environmental fluctuations caused by climate change [9, 10, 11, 12].

Oxidative stress is becoming an increasingly important process to include in ecological work, studying global warming and other human-induced environmental changes [13, 14]. Warming, UV-B radiation, hypoxia and salinity fluctuations can cause oxidative stress in animals inhabiting the marine environment [15, 16, 17], causing an imbalance between the reactive oxygen species (ROS) and its elimination [18, 19]. In case an imbalance occurs between the level of ROS and antioxidant protection, it can result in oxidative damage to tissues and a state of oxidative stress [20]. Baltic Sea zooplankton is reported to be sensitive to both chemical contamination and changes in oxygen and temperature. Vehmaa et al. [21] found that a 3 degrees rise in temperature increased the antioxidant capacity (ORAC, Oxygen Reactive Absorbance Capacity) in *Acartia* copepods by almost 15%, and they measured a 2-fold increase also in oxidative damage, measured as lipid peroxidation. When involving also other stressors such as pH and toxic algae, a decrease in antioxidant was detected [21]. The glacial relict copepod *Limnocalanus macrurus*’ antioxidant defense and oxidative stress biomarkers showed associations with hydrographic factors and selected proxies describing organochlorine contaminant loads during a large survey covering the northern Baltic Sea basins [16]. Nevertheless, toxic cyanobacterial blooms can also increase antioxidant defense levels and improve oxidative status, as Hogfors et al. [22] showed in their work reporting ORAC: TBARS ratio (TBARS, measuring lipid peroxidation) in *Acartia bifilosa*. In addition, some organisms can protect themselves from oxidative damage, an event called “preparation for oxidative stress” [23], as shown in the mysid shrimps of the genus *Mysis*, which seem fairly tolerant to large variation in dissolved oxygen concentrations [17].

In terms of climate change, the coastal environment is essential to study due to the high variability of hydrographical variables commonly monitored in aquatic studies, such as temperature, nutrients, chlorophyll *a* (Chl *a*), pH and dissolved inorganic carbon (DIC). The Baltic Sea, in which the present study is implemented, is especially sensitive to environmental change as the area is heavily eutrophicated and suffers from large-scale deep-water anoxia [24], enhanced by global warming [25].

The main aim of the study was to obtain a more comprehensive understanding of the oxidative stress status in copepods as they experience large fluctuations in their biotic and abiotic environment on a daily basis. Moreover, copepods are known to respond on a short-time scale to fluctuations in environmental conditions in the water column [26, 27]. For this purpose, we based our study on one of the most abundant zooplankton species *Acartia* sp., which were collected from different depths (according to the depth of maximum copepod abundance) and times of day (night and day) during a two-week field survey in the western Gulf of Finland. We measured different biomarkers to reveal potential relationships between copepod antioxidant defense, oxidative stress and the environmental condition (Chl *a*, pH, DIC, salinity, temperature) at different depths. Finally, this study will help to increase our understanding concerning the cellular responses to climate change in this species.

## Material and methods

### Sampling

The sampling was carried out during two weeks in August 2015 at Storfjärden monitoring station (59° 52' 56" N, 23° 15' 14" E) in the western Gulf of Finland of the Baltic Sea. In 2015, water temperature varied between ~1°C in January-February and 17.5°C in August. Salinities ranged between 5.64 and 6.46 with higher salinities in deeper waters. pH values fluctuated between 7.57 and 8.57 with the highest value observed in August in the layer 0-10m (S1 Fig). The copepods were sampled with a 150 μm closing net at specific depths (in accordance with maximal distribution of copepods [7]) and time of day (Table 1). After each haul (5 per sampling time), the animals were immediately transferred into coolers to 20 L of seawater from the sampling depth. After returning to the laboratory, the samples were stored in a climate chamber until sorted for picking (<6 h in total). In this study, our main target species was adult copepods *Acartia bifilosa*, the most abundant copepod species in the area. However, we cannot exclude that some of the specimens collected were *Acartia tonsa*, which occurs in the community in late summer (hereafter *Acartia* sp.). The number of replicates was dependent on the copepod abundance at the considered depth. Therefore, as we usually managed to collect 3–4 replicates, only one replicate was collected on the 11 August 2015 (Table 1). Thirty adults *Acartia* (size <1 mm) were rapidly sorted on ice individually using forceps, and transferred to a 2 mL plastic Eppendorf microtube. Samples were stored immediately in -80°C until analyses [21]. A comparison between samples frozen in -80°C, and samples snap-frozen in liquid N and stored in -80°C were made, to ensure there were no differences caused by methodology (t-test, p > 0.05).

**Table 1:** Main hydrographic variables measured (mean ± SD) and the number of zooplankton samples collected during the study. Weeks I and II are separated by a thick border.

Date	Sampling Time	Sampling depth (m)	T°C (mean ± SD)	Salinity (mean ± SD)	Oxygen (mg l^-1^)	pH	Number of zooplankton samples
10.08.2015	12:00	20–30	10.18 ± 0.67	6.03 ± 0.02	6.50 ± 0.31	7.37	3
11.08.2015	18:00	10–20	13.08 ± 0.74	5.98 ± 0.01	8.60 ± 1.30	7.84	1
13.08.2015	00:00	0–10	16.70 ± 2.17	5.76 ± 0.27	10.40 ± 0.90	8.02	3
14.08.2015	06:00	15–25	11.30 ± 0.89	5.99 ± 0.02	7.12 ± 1.15	7.48	3
17.08.2015	12:00	20–30	12.08 ± 0.28	6.00 ± 0.01	6.83 ± 0.75	7.84	4
18.08.2015	18:00	10–20	15.62 ± 1.47	6.02 ± 0.08	9.22 ± 1.21	8.21	3
20.08.2015	00:00	0–10	17.96 ± 0.47	5.94 ± 0.29	11.43 ± 0.90	8.52	3
21.08.2015	06:00	15–25	14.66 ± 1.04	5.94 ± 0.02	8.16 ± 1.37	7.77	4

### Hydrography

Dissolved oxygen (DO) was measured in the field at 0, 5, 10, 15, 20, 25 and 30 m using an oxygen meter (ProDSS Sensor, YSI Incorporated, Yellow Springs, OH, USA, resolution 0.1, accuracy 0.1 mg L^-1^). The water samples for pH, Chl *a* and DIC analyses were collected at 5, 15 and 25 m depth using a 2 L Limnos water sampler. For pH analysis, seawater was sampled in 250 mL airtight glass bottles and kept in a cooler on board. The Jenway 3510 pH meter (Bibby Scientific, Ltd., Staffordshire, United Kingdom) was calibrated daily using pH buffers 4.0, 7.0 and 10.0, and sensor kept in pH 4.0 after calibration. The samples were measured after reaching room temperature (~20°C). Chl *a* analysis was determined according to Almén et al. [7]; samples were collected in 1000 mL plastic bottles and protected from light after sampling. In the laboratory, 100 mL was filtered on a Millipore vacuum filtration device using glass-fibre filters (25 mm, GF/F Whatman). The samples were dissolved with 5 mL ethanol (96%) in the dark for 24 h, and Chl *a* was determined by fluorometry (Varian Cary Eclipse Fluorescence Spectrophotometer, Varian Optical Spectroscopy Instruments, Mulgrave, Australia), using a 96-well microplate reader. Samples for total DIC were collected in triplicate from each of three depths into 25 mL acid-washed, airtight glass bottles, stored in dark on ice at +3°C until measured within 12 h, using the acidification/gas stripping/infrared detection method [11, 28]. Temperature, salinity and conductivity were measured with a CTD probe (Valeport Mini CTD, Valeport Limited, Devon, United Kingdom) from the surface to the bottom.

### Determination of biomarkers

A number of biomarkers (described in Table 2) was assessed to reveal responses in antioxidant defense and oxidative stress of *Acartia* sp. to environmental conditions. Zooplankton samples (thirty adults *Acartia* sample^-1^) were entirely homogenized in 100 μL of 0.1 M K_2_HPO_4_ + 0.15 M KCl buffer (pH 7.4) using a Tissue Lyser II bead mill (Qiagen). An aliquot of raw homogenate (25 μL) was immediately frozen in liquid nitrogen and stored at -80°C for lipid peroxide determination (LPX). Then, the sample homogenate was centrifuged at 10,000g for 15 min at 4°C and the resulting supernatant was divided into aliquots for Glutathione S-transferase (GST), Glutathione reductase (GR), Catalase (CAT) and Superoxide dismutase (SOD) enzyme activity determination, Oxygen Radical Absorbance Capacity (ORAC) assay and for glutathione sample preparation.

**Table 2:** Definition, justification, interpretation of used biomarkers with zooplankton examples from literature. AO = antioxidant, OD = oxidative damage, O_2_^-^ = superoxide radical, H_2_O_2_ = hydrogen peroxide.

	Biomarker	Definition	Justification	Interpretation ↑ or ↓	Example of species and study area	Reference
GSH: GSSG	Reduced: oxidised glutathione ratio	OD	Assays glutathione redox state [29]	Low ratio or increase in GGSG indicate stress	*Limnocalanus macrurus*, Baltic Sea, FI	[16]
GR	Glutathione reductase	AO	Reduces GSSG back to GSH [30]	↑ indicates more AO	*L*. *macrurus*, Baltic Sea, FI	[16]
GST	Glutathione S-transferase	AO	Catalyzes reactions that detoxify harmful compounds [31]	↑ indicates more AO	*L*. *macrurus*, Baltic Sea, FI; *Eurytemora affinis*, Seine estuary, FR	[16, 27]
SOD	Superoxide dismutase	AO	Part of enzymatic defense to remove O_2_^-^ [31]	↑ indicates more AO	*L*. *macrurus*, Baltic Sea, FI	[16]
CAT	Catalase	AO	Part of enzymatic defense to remove H_2_O_2_ [31]	↑ indicates more AO	*Boeckella gibbosa*, *Mixodiaptomus laciniatus*, Lake Los Cantaros, ARG; Lake La Caldera, ESP	[32]
ORAC	Oxygen reactive absorbance capacity	AO	Assesses antioxidant capacity [33]	↑ indicates more AO	*Acartia bifilosa*, Baltic Sea, FI	[21]
LPX	Lipid peroxidation	OD	Oxidative degradation of lipids in cell membrane, resulting in cell damage [33]	↑ indicates stress	*L*. *macrurus*, Baltic Sea, FI	[16]

The Total GSH sample was deproteinized by adding 5% sulfosalicylic acid (SSA) and subsequently incubated on ice for 10 min and centrifuged for 10 min at 10,000g at 4°C. The supernatant was divided into two different tubes for reduced (GSH) and oxidized glutathione (GSSG) and 33 mM M_2_VP (1-methyl-2-vinylpyridinium trifluoromethanesulfonate, Sigma Chemicals) in 0.1M HCl, a scavenger of GSH, was added to the GSSG sample. The sample homogenate aliquots and glutathione samples were frozen in liquid nitrogen and stored at -80°C until further analysis. GST and GR activities were determined as described in Vuori et al. [16]. Glutathione 384-well plate Fluorescent Detection Kit (Arbor Assays) was used to measure GSH and GSSG fluorescent emission at 510 nm, with excitation 370–410 nm. The FOXII assay, modified from the protocols in Bou et al. [34] and Eymard et al. [35], was used to measure lipid peroxidation (LPX) in the samples, as described in Vuori et al. [16]. The raw homogenates were mixed with methanol and centrifuged at 3,000g for 5 min at room temperature. FOXII reagent (450 μL) was added to the samples (50 μL), and absorbance measured at 590 nm after 2 h of incubation. The CAT activity measurement was modified from the Catalase Assay kits’ colorimetric assay (Sigma Chemicals) to a 96-well microplate, as described in Vuori et al. [36]. The reaction of catalase and H_2_0_2_ (total volume 7.5 μL, containing 0.133 μg μL^-1^ sample protein) was stopped with NaN_3_. Two μL of the reaction was pipetted to a microplate and the leftover H_2_O_2_ was detected with colorimetric reaction by adding 200 μL of color reagent to the wells, incubating for 15 min and measuring the absorbance at 520 nm. The inhibition rate of SOD was measured with the SOD determination kit (Sigma Chemicals). Intracellular soluble antioxidant capacity was measured with OxiSelect^TM^ Oxygen Radical Antioxidant Capacity (ORAC) Activity Assay (Cell Biolabs) following the manufacturers’ instructions, except for adjusting the reaction volumes for 384-well plate, when needed. The enzyme activities, lipid hydroperoxides and total GSH were normalized to the protein content of the samples, which were determined with Pierce^TM^ BCA Protein Assay (Thermo Scientific) with bovine serum albumin (Sigma) as the standard.

All samples, standards and blanks were analyzed in triplicate, and a positive control was included to ensure the assay worked as expected. For all assays performed in this study, the mean coefficient of variation percentage (CV%) of technical replicates ranged between 1.69 and 6.24%.

### Statistical analyses

All data were tested for normal distribution using the Shapiro-Wilk normality test. The environmental data were not normally distributed and were analyzed using the k-sample Kolmogorov–Smirnov test. Differences between different samples storage methods were analysed with Independent sample t-test. Differences of environmental parameters between weeks and depths were tested using General Linear Model (GLM). The biomarker variables that were not normally distributed were log transformed. All biomarkers (response variables) were analyzed in association with the environmental conditions using a Linear Mixed Model (LLM), fitted by REML (Restricted Maximum Likelihood) estimation using the package ‘lmerTest’ in R [37]. The Welch-Satterthwaite approximation was used for p-values and degrees of freedom. Environmental variables were used as fixed factors and sampling occasion (date) was added as random factor. Some missing values were replaced with the group mean (1/25 GST and SOD values, 2/25 ORAC and 2/24 CAT values). A three-table ordination method, RLQ analysis [38] was used for visualizing the associations between measured antioxidant defense and oxidative stress variables in the samples and environmental data. First, PCA principal component analysis was done for both the biomarkers and the environment data, and then a correspondence analysis on the sample groups. GR and GSSG were indicated as present (P) when detected, and absent (A) when under detection level. These three analyses were then passed to the RLQ function of ‘ade4’ package in R [39]. SPSS 21.0. and the free statistical software R, version 3.2.3, were used for the data analyses.

## Results

### Hydrography and environmental parameters

The different variables measured during the present study varied between sampling depths and dates (Table 1). Neither thermocline, nor halocline was observed between 0 and 30 m depth. Temperatures were higher at the surface, and increased by almost 2°C in all depths between Weeks I and II (Table 1). Salinity varied between 5.76 ± 0.27 at 5 m depth and between 6.03 ± 0.02 at ~25 m. Dissolved oxygen concentrations showed a gradient from surface to deep water (~25 m). pH decreased with depth and ranged between 8.02 and 7.37 in Week I, and between 8.52 and 7.84 in Week II. Water temperature and salinity differed significantly between weeks and between depths (GLM, p-values < 0.001). Oxygen differed between depths (GLM, p-values < 0.001), whereas neither oxygen nor pH differed between weeks (GLM, p-values > 0.05).

Weather conditions were stable during Weeks I and II with clear (night time) or sunny skies. Winds were below 1m s^-1^ (personal observation).

### Oxidative stress status

The oxidative stress status of the *Acartia* population was analysed using different biomarkers for antioxidant defense (GR, GSH, GST, CAT, SOD and ORAC), glutathione oxidation (GSSG) and oxidative stress (LPX, Tables 2 and 3).

**Table 3:** Antioxidant defense and oxidative stress variables measured in *Acartia s*p. in August 2015. Week I and II are separated by a thick border; CAT: Catalase, ORAC: Oxygen radical absorbance capacity, SOD: Superoxide dismutase, GR: Glutathione reductase, GST: glutathione S-transferase, totGSH: total glutathione, GSSG: oxidized glutathione, LPX: Lipid peroxidation, ND: under detection level, n: number of samples.

			Antioxidant defense						Oxidative stress	
Date	Depth (m)		CAT (μmol min^-1^ mg^-1^)	ORAC (μM trolox equivalents mg^-1^)	SOD Inhibition (%)	GR (nmol min^-1^ mg^-1^)	GST (μmol min^-1^ mg^-1^)	totGSH (μM mg^-1^)	GSSG (μM mg^-1^)	LPX (μM cumene-hydroperoxide equivalents mg^-1^)
10 Aug.	20–30		7.59 ± 3.06	323.87 ± 168.32	57.65 ± 6.56	1.49	0.15 ± 0.04	26.86 ± 5.62	ND	33.49 ± 9.15
		n	2	3	3	1	3	3		3
11 Aug.	10–20		98.10	179.72	69.42	ND	0.20	33.85	ND	53.99
		n	1	1	1		1	1		1
13 Aug.	0–10		23.77 ± 2.08	353.21 ± 318.02	69.98 ± 4.59	1.69	0.21 ± 0.08	21.46 ± 2.02	ND	30.74 ± 7.09
		n	3	3	3	1	3	4		4
14 Aug.	15–25		17.69 ± 5.71	67.14 ± 23.32	59.23 ± 4.71	ND	0.09 ± 0.07	55.49 ± 24.46	3.81 ± 0.13	37.26 ± 5.38
		n	3	3	3		3	3	2	3
17 Aug.	20–30		17.69 ± 5.14	122.50 ± 23.46	53.17 ± 13.57	1.41	0.15 ± 0.04	27.83 ± 16.66	3.53	39.89 ± 11.59
		n	4	4	4	1	4	4	1	4
18 Aug.	10–20		13.75 ± 1.82	172.60 ± 99.50	50.45 ± 3.59	0.07	0.24 ± 0.01	60.42 ± 5.22	3.96 ± 0.49	29.75 ± 9.25
		n	3	3	3	1	3	3	3	3
20 Aug.	0–10		15.75 ± 5.23	128.26 ± 75.77	41.18 ± 3.98	4.12 ± 1.28	1.14 ± 0.54	38.34 ± 4.91	4.24 ± 0.33	30.22 ± 8.76
		n	2	3	3	3	3	3	3	3
21 Aug.	15–25		15.87 ± 4.33	146.63 ± 30.11	44.79 ± 11.40	4.56 ± 1.00	0.24 ± 0.06	41.65 ± 17.41	3.59 ± 2.04	34.08 ± 10.67
		n	4	4	4	3	4	4	4	4

**Fig 1:**
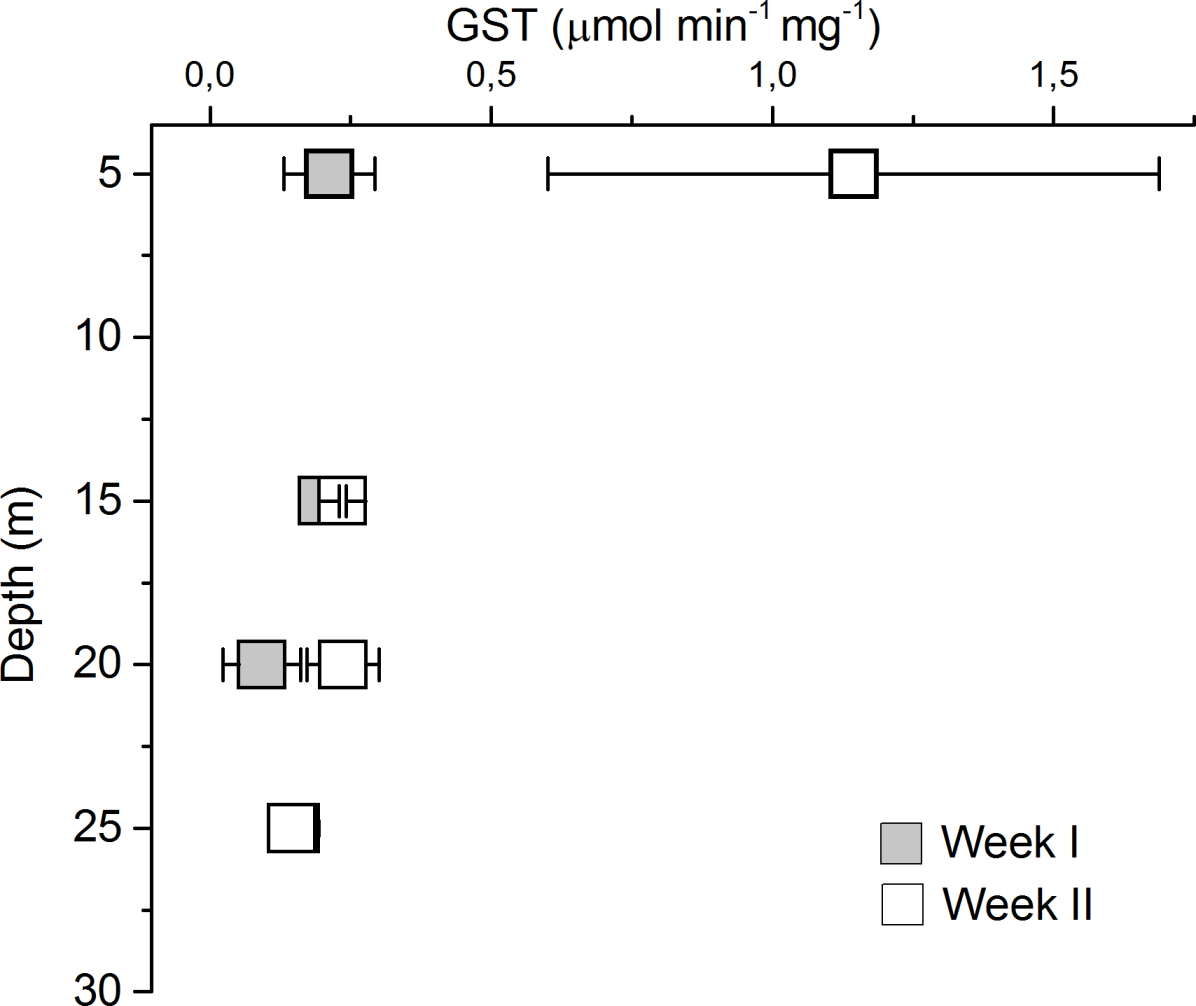
Glutathione S-transferase activity measured in *Acartia* sp. The animals were collected at different depths (5, 15, 20 and 25 m) during two weeks in August 2015.

The catalase activity was quite low with values below 23.77 μmol min^-1^ mg^-1^ except at 15 m in Week I when the activity was 98.10 μmol min^-1^ mg^-1^. ORAC activities were found to be higher during Week I, except at 20 m. The SOD activity was higher in Week I (~60%) compared with Week II (~50%). GST activity was six-fold higher during Week II in the surface compared with other depths during both weeks (Table 2, Fig 1). The GR activity was below limit of quantification (≤ 0.07 nmol min^-1^ mg^-1^) for 58% of the samples especially in Week I, whereas GR was detected in the end of Week II (Table 2). Lipid peroxidation (LPX) was highest in *Acartia* at 15 m depth during Week I (53.99 μM mg^-1^ protein) and lowest during Week II at the same depth (29.75 ± 9.25 μM mg^-1^ protein).

The oxidized form of glutathione (GSSG) was below limit of quantification (≤ 0.10 μM mg^-1^) during most of Week I, except at 20 m, while GSSG was detected during Week II (Table 2).

### Biomarkers and environmental variables

The GST activity was found to be higher in samples collected when temperature (LMM, t _5.95500_ = 3.425, p = 0.014), oxygen (LMM, t_5.9770_ = 3.281, p = 0.017) and pH (LMM, t_6.0710_ = 3.815, p = 0 .009) were higher. A higher level of LPX was found to be associated with lower Chl *a* concentration (LMM, t_23.000_ = -2.307, p = 0.030), and lower salinity with higher CAT activity (LMM, t_4.487_ = -3.026, p = 0.034). No significant relationships were found for ORAC, SOD, total GSH and environmental variables.

**Fig 2:**
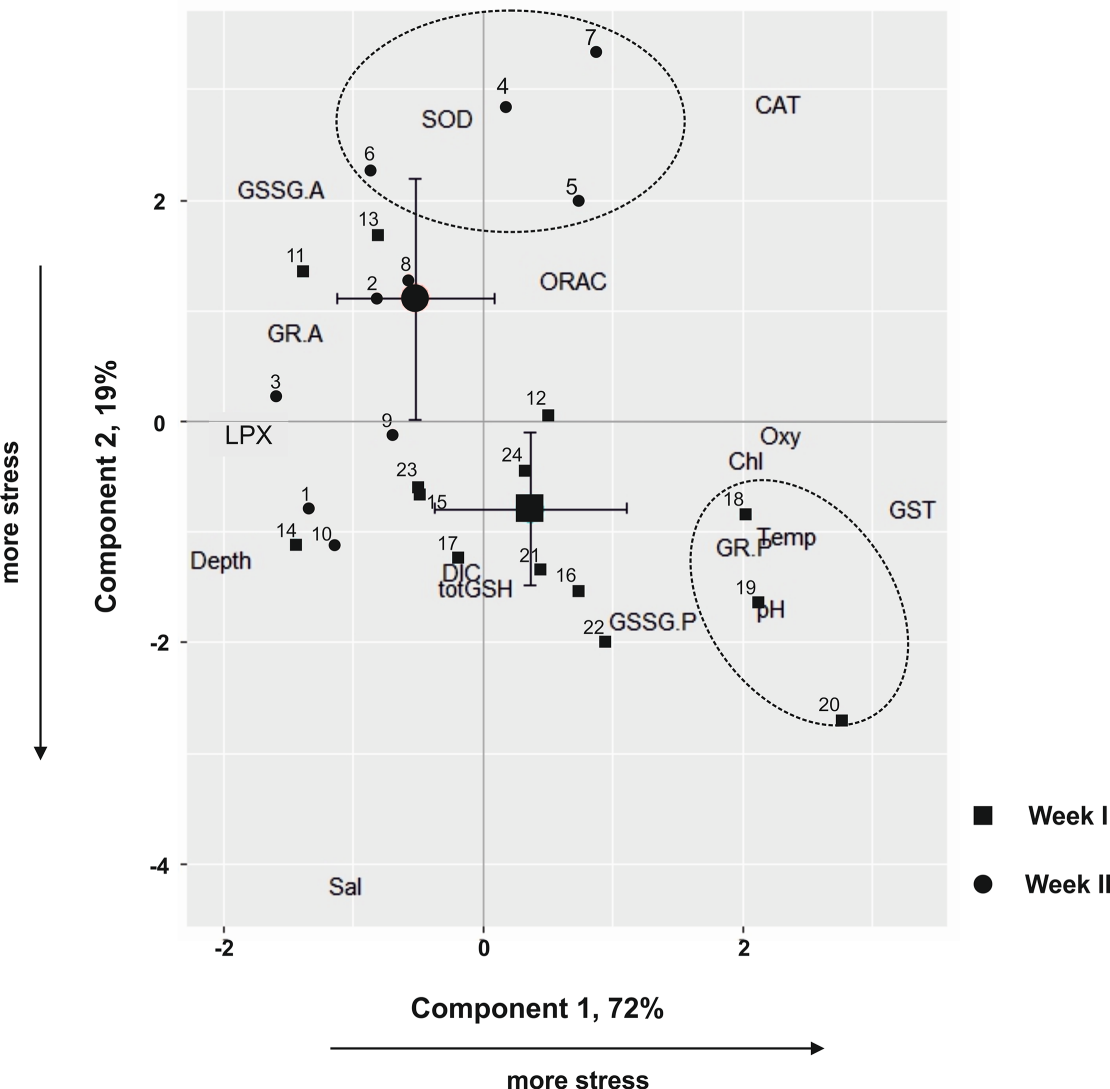
Component 1 and 2 scores from Principal Component Analysis (PCA) for *Acartia* sp. collected in the western Gulf of Finland during two weeks. Samples collected during Week I (black dots 1–10), and samples collected during Week II (black squares, 11–24). The corresponding loading plots (biomarker variables and environmental variables) are superimposed on the scores plot. Abbreviations are as follows: CAT Catalase, LPX Lipid peroxidation, GST Glutathione S-transferase, ORAC Oxygen radical absorbance capacity, SOD Superoxide dismutase, totGSH total glutathione, GSSG.A oxidized glutathione Absent, GSSG.P oxidized glutathione Present, GR.A Glutathione reductase Absent, GR.P Glutathione reductase Present. One sample from 11 August was not considered in the PCA analysis as there were no replicates.

In the PCA (Fig 2, Table 4), GST, and GR.P (P = present) were associated with Component 1, whereas LPX and GR.A (A = absent) had an inverse relationship with these variables. Component 2 was positively associated with CAT, ORAC, SOD and GSSG.A and negatively associated with total GSH and GSSG.P. The two main components explained in total > 90% of the observed variation in the PCA, i.e., 72.33% of Component 1 and 18.99% of Component 2. Samples from Weeks I and II were clearly separated in the PCA score plots (Fig 2). High GST and total GSH values were recorded in *Acartia* during Week II, whereas specimens collected during Week I exhibited higher levels of SOD activities (Fig 2). This tendency was especially true for samples collected at the surface. Activity in both GR and GSSG were detected in samples in Week II, but not during Week I, whereas lipid peroxidation LPX levels were higher in Week I and lower in Week II. The increased CAT activity was associated with lower salinity and vice versa (Fig 2).

**Table 4:** Component matrix for Fig 2. (A). Biomarker abbreviations are as follows: CAT: Catalase, LPX: Lipid peroxidation, GST: glutathione S-transferase, ORAC: Oxygen radical absorbance capacity, SOD: Superoxide dismutase, totGSH: total glutathione, GSSG.A: oxidized glutathione Absent, GSSG.P: oxidized glutathione Present, GR.A: Glutathione Reductase Absent, GR.P: Glutathione Reductase Present. (B). Environmental variables. Loadings highlighted in bold represent the highest absolute value of the parameter when considering different PCs.

**(A)**	**Component**	
	**1**	**2**
GST	**0.663**	-0.156
GR.P	**0.404**	-0.226
LPX	**-0.364**	-0.019
GR.A	**-0.288**	0.162
CAT	0.456	**0.575**
SOD	-0.051	**0.549**
GSSG.A	-0.309	**0.423**
GSSG.P	0.262	**-0.358**
totGSH	-0.008	**-0.298**
ORAC	0.141	**0.257**
**(B)**	**Component**	
	**1**	**2**
Temperature	**0.469**	-0.206
Oxygen	**0.458**	-0.022
pH	**0.445**	-0.337
Chlorophyl a	**0.406**	-0.069
Depth	**-0.401**	-0.249
Salinity	-0.211	**-0.839**
DIC	-0.030	**-0.270**

## Discussion

The site in the western Gulf of Finland, where the copepods of the genus *Acartia* were sampled for oxidative stress and antioxidant defense measurements, is characterized by a strong thermocline in late summer, shown as low temperatures and low pH at the bottom, but also occasionally higher salinity, due to seasonal upwellings. In the present study (August 2015), no sharp clines were found. However, the temperature elevated by almost 2°C during the two-week field survey and the study area also suffered from cyanobacteria blooms (mainly *Aphanizomenon* and *Nodularia*, personal observation).

We showed that higher temperature, pH and oxygen were associated with increased activities of Glutathione S-transferase (GST) in *Acartia* sp., which is among the first enzymes to respond to stressors, such as temperature [27]. GSTs are multifunctional proteins, being important phase II enzymes [40], and that in association with Glutathione (GSH) transform xenobiotics (i.e., foreign elements) into other conjugates as part of a detoxification route, playing major roles in the metabolic ‘purification’ and in the defense against ROS [41, 42]. Considering time of response, GSTs are known to react on a short-time scale (within an hour) to both temperature and salinity in copepods [27]. The antioxidant defenses (i.e., GST, CAT, SOD) are known to activate due to warming, both in cladocerans [43, 44], mussels [45] and crabs [46]. Moreover, Vehmaa et al. [21] showed that a rise by 3°C was affecting copepods’ oxidative balance by increasing ORAC and TBARS levels, whereas toxic cyanobacteria promoted antioxidative defenses (ORAC) and decreased oxidative damage (TBARS). In the amphipod *Gammarus*, Turja et al. [47] measured elevated antioxidative defenses (GST, GP glutathione peroxidase and SOD) as a response to nodularin, a common cyanobacteria toxin in the Baltic Sea. In our study area, surface pH and oxygen increased (Table 1), most likely due to microalgae (Chl *a* and cyanobacteria increased, personal observation), taking up DIC for growth and photosynthesis [48, 49]. The high dissolved oxygen (DO) concentrations during Week II (from 6.83 ± 0.75 to 11.43 ± 0.90 mg l^-1^, in deep layers and the surface, respectively) could also have influenced GST to respond. Our results are supported by measurements from the glacial relict *Limnocalanus macrurus*, exhibiting higher GSTs in elevated DO concentration [14]. Hermes-Lima et al. [50] also suggest that redox-sensitive transcription factors and antioxidant defense are activated under low oxygen levels or even hypoxia, in order to prepare the organisms for oxidative stress during reoxygenation phase, in which a sharp overproduction of ROS is expected to take place. Despite fast changing conditions [51], copepods experience widely varying conditions in their physico-chemical environment during diel vertical migration [7]. Nevertheless, vertical migration is an important way of escaping harmful conditions, as shown in euphausiid krill [42], whose redox status is positively affected by migration [52]. ORAC and SOD did not respond to environmental variables, and considering that they were fluctuating on a weekly basis, it suggests that the copepods were already facing oxidative stress, potentially as a response to differential allocation of resources to growth or reproduction. When antioxidants rise it indicates either a decrease in reproduction with no accelerating senescence, or a reduced investment in functions, such as immunity, causing increased risk of mortality [18]. In addition, CAT activity had an inverse relationship with salinity, with higher activity when the salinity was low and vice versa (Fig 2). This response in *Acartia* is interesting as the salinity changes in our study site are relatively small (Table 1). Whitfield et al. [53] showed that salinities 5–8 are critical for brackish-water animals inhabiting low salinity areas, including that even small salinity changes can be important for animal’s general performance. In the mud crab *Scylla serrata*, increasing salinity (from 10 to 35) induces an increase and/or decrease in the antioxidant enzyme activities depending on the tissue (abdominal muscle, hepatopancreas, or gills) considered [54]. In the white shrimp *Litopenaeus vannamei*, the SOD and CAT activities were increased at low salinity (3‰) in both the muscle and the hepatopancreas [55].

The oxidized glutathione (GSSG), which is an indication of oxidative stress, is generally maintained at a level of 1–10% of the total glutathione through reduction by glutathione reductase (GR) [56]. In the current work, the GSSG level was below the limit of quantification during most of Week I (except at 20 m), whereas it was detected during Week II, suggesting increased oxidative stress, due to changes in hydrography between Weeks I and II. In line with increased GSSG level and oxidative stress, GR activity was detected during Week II in the surface and at 15–25 m depth, and suggests oxidative stress in *Acartia*, as GR is activated upon accumulation of GSSG [57]. We measured elevated temperature in the surface during Week I, which probably caused increased antioxidants, in general (Table 3, 0–10 m). In terms of oxidative damage, differences in LPX levels between both weeks can at least partially be explained by increasing GST during Week II, as this enzyme is known to metabolize organic hydroperoxides by using reduced glutathione [58, 59]. Moreover, we showed that *Acartia* copepods residing in the deep during daytime responded by elevated LPX activity when the Chl *a* concentration was low. We are not totally sure what is the reason behind rising LPX; low food quantity and/or low food quality could be factors to consider. Long-term elevated oxidative stress status is harmful for the animal activity level, health status and general condition, and can result in decreased growth, reproduction, early ageing and even death [13]. Studies in Chesapeake Bay reported high numbers of dead *Acartia* [60], and the question arises whether copepod mortality is high, in general, in turbulent estuaries, or if the environmental quality of the site was bad [51]. Also in the current study site, *in situ* mortality of *Acartia* varies between 10–40% [61]. LPX is known to correlate with high age and mortality [62], and can cause both protein and ion channel inactivation, as well as protein damage through generation of reactive aldehydes, resulting in secondary protein carbonylation [63]. One effect of LPX is to increase membrane fluidity by introducing hydro peroxide groups, which open pores in the membrane causing leakiness, allowing substances to enter that do not normally pass it otherwise than via specific channels [64]. The oxidative stress levels can also be affected by nutritional conditions, because some of the antioxidant defenses are acquired from the food source [18]. The most important antioxidants are considered to be vitamin E and various carotenoids [65]. However, there is some contradicting opinions on how important dietary antioxidants are for combating ROS (cf. Monaghan et al. [18]), as some studies have over-emphasized their importance for ROS destruction [66]. Rodríguez-Graña et al. [67] and Saiz et al. [62] demonstrated that ageing copepods exhibit lower feeding and egg production, and higher oxidative damage rates than younger copepods. Even though we did not measure feeding and reproduction in the present study, ageing is a potential factor bringing variability into the results, and it is most likely a factor to be considered in further studies. We do not have age data of the copepods in the current work, sampled from a wild population, which probably consisted of individuals of various age. It also means that we collected copepods with different degrees of maturation and development of gonads, a process which is known to be associated with changes in biochemical composition, including levels of lipid peroxidation and antioxidants (see [68, 69] and references therein). Apart from ageing, another factor that may add to the variability of the results is the background (i.e., previous life history) of the copepods. In addition, salinity, both temperature, pH and oxygen varied substantially during the study. Considering that fluctuations keep the acclimation process up in animals residing in the natural environment [70], one must note that we do not account for how large the fluctuations were prior to the work in the present study.

## Conclusions

In the present study, we found that copepods were quite robust in regards to pH encountered in the deep layers, and to values, similar as expected in oceans by the end of this century. This finding is in accordance with other studies showing that copepods seem fairly tolerant to hydrographical coastal variability. Copepods perform vertical migration on a daily basis, and could be adapted to extremely variable environment. One of these adaptations could be a highly efficient glutathione cycling system, functioning as an antioxidant defense system for neutralizing ROS and avoiding elevated levels of LPX. However, GST activities responded to elevated temperature, oxygen and pH experienced by the animals near the surface of Week II, suggesting that copepods were enhancing their antioxidant capacity to counterbalance the harmful effect of reactive oxygen species produced in response to warming and algal growth. This study highlights the importance of evaluating the antioxidant defense, as well as the oxidative stress in copepods. Future studies should consider the effect of environmental stressors on the trade-off between oxidative balance and reproductive success of *Acartia*.

## Supporting information

S1 Data Antioxidant defense and oxidative stress variables measured in *Acartia* sp. in August 2015 (raw data). (XLSX)

S1 Fig Mean temperature, salinity and pH recorded at Storfjärden monitoring station in 2015. (TIF)
