# Supplementary figures and images for: Oxidative stress and antioxidant defense responses in Acartia copepods in relation to environmental factors
Source: PLoS One. 2018 Apr 13;13(4):e0195981. doi: 10.1371/journal.pone.0195981 (PMC5898752; doi:10.1371/journal.pone.0195981)

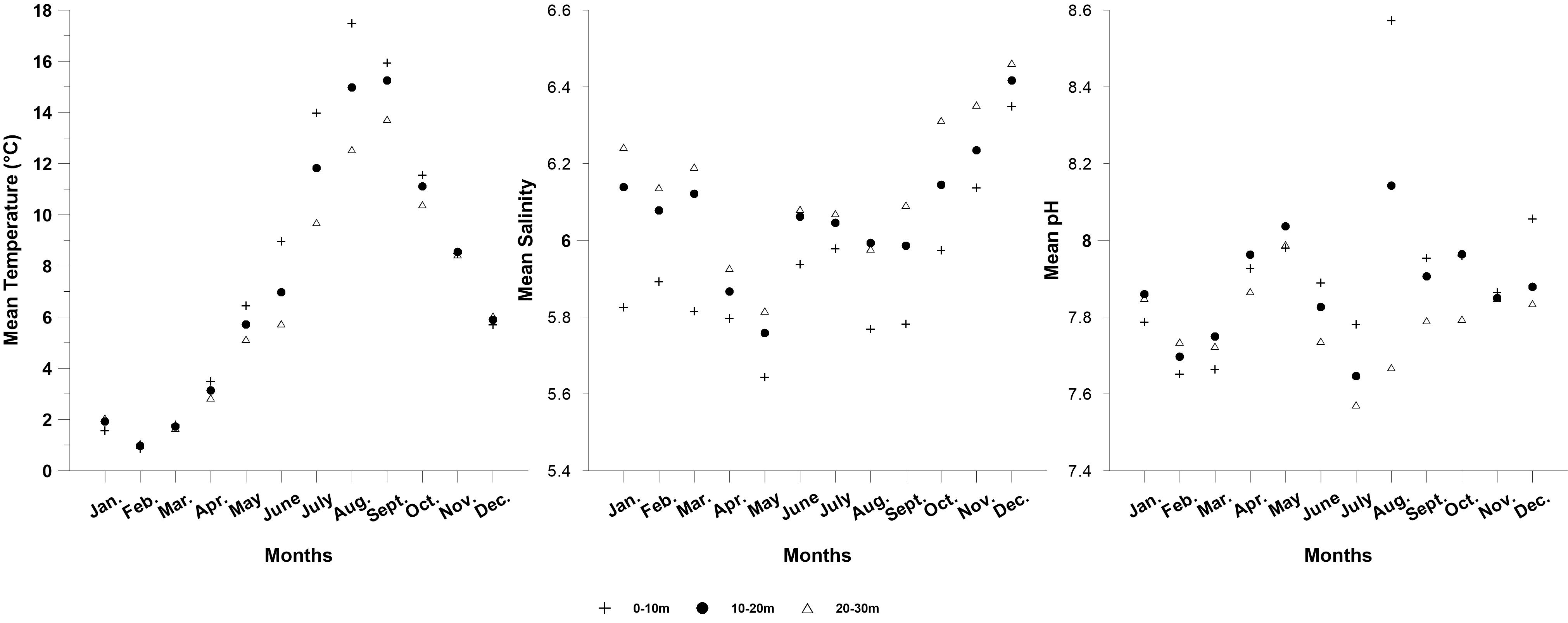

Supplement: S1 Fig — (TIF) [file pone.0195981.s002.tif]
